# Anti-Atherogenic Activity of Ethanolic Fraction of *Terminalia arjuna* Bark on Hypercholesterolemic Rabbits

**DOI:** 10.1093/ecam/neq003

**Published:** 2011-03-20

**Authors:** Saravanan Subramaniam, Ramachandran Subramaniam, Suja Rajapandian, Subasini Uthrapathi, Victor Rajamanickam Gnanamanickam, Govinda Prasad Dubey

**Affiliations:** ^1^Department of Pharmacology and Toxicology, PSG College of Pharmacy, PSG Institute of Medical Science and Research, Peelamedu, Coimbatore 641 004, India; ^2^Department of Pharmacology, KMCH college of Pharmacy, Coimbatore, India; ^3^Department of Biochemistry, PRIST University, India; ^4^Department of Pharmacology, CARISM—Pharmacy, SASTRA University, Thanjavur, Tamil Nadu, India

## Abstract

Atherosclerosis which results from gradual deposition of lipids in medium and large arteries is a leading cause of mortality worldwide. *Terminalia arjuna* is a herb of Combretaceae family which contains hypolipidemic compounds and flavonoids with high antioxidative properties. This study was conducted to determine the effect of ethanolic fraction of *T. arjuna* on blood lipids and atherosclerosis in rabbits fed with high fat diet (HFD). Twenty New Zealand rabbits of either sex were randomly divided into five groups: the first two were normal diet group and HFD (21% fat) group and the remaining three groups received high cholesterol diet supplemented with standard drug (Atorvastatin 10 mg kg^−1^ body weight), *T. arjuna* ethanolic fraction (100 and 200 mg kg^−1^ body weight), respectively. The concentration of total cholesterol (TC), low density lipoprotein (LDL) cholesterol, triglycerides (TGs), very low density lipoprotein (VLDL) cholesterol and high density lipoprotein (HDL) cholesterol was determined in rabbits at the start of the experiment, at the 14th, 30th days and at the end of the study. Anti-atherogenic index was calculated from the lipid profile of the rabbits before sacrifice. At the end of the experimental period, the aorta was removed for assessment of atherosclerotic plaques. Results show that *T. arjuna* significantly decreases TC, LDL and TG levels and increases HDL and lessens atherosclerotic lesion in aorta (*P* < .05). Hence *T. arjuna* extract can effectively prevent the progress of atherosclerosis. This is likely due to the effect of *T. arjuna* on serum lipoproteins and its antioxidant and anti-inflammatory properties.

## 1. Introduction

Atherosclerosis leading to coronary artery disease has assumed a virulent disease ratio and is the principal cause the world over in the developed as well as in the developing countries. Epidemiologic studies have demonstrated a positive significant relationship between plasma cholesterol concentrations and coronary artery disease [1]. Clinical manifestations are plaque formation in the artery walls. In many cases, plaques protrude into the lumen of the artery and if sufficiently large, compromise the flow of blood [2]. Synthetic anti-hyperlipidaemic drugs like statins and synthetic antioxidants like probucol are widely used to treat atherosclerosis. Unfortunately, these drugs have side effects [3]. Hence, for treatment of atherosclerosis, much attention has been focused on the use of natural products that have very few side effects [4]. One such plant is *Terminalia arjuna* (Family: Combretaceae), used in traditional medicine for treating ulcers [5], wound healing [6], and also for antibacterial [7], antimutagenic/anticarcinogenic [8, 9], antioxidant and hypocholesterolemic activities [10]. The active constituents of *Terminalia* include tannins, triterpenoid saponins (arjunolic acid, arjunic acid, arjungenin, arjunglycosides), flavonoids (arjunone, arjunolone, luteolin), gallic acid, oligomeric proanthocyanidins (OPCs), polyphenols, calcium, magnesium, zinc and copper [11]. Prolonged administration of *T. arjuna* did not show any adverse effect on renal, hepatic and hematological parameters [12]. But the fraction type of *T. arjuna* bark having a high degree of antioxidant, anti-hyperlipidaemic and anti-atherogenic activity is not well known. The efficacy of *T. arjuna* fractions as antidyslipidemic and antioxidant agents was found as: ethanolic fraction > diethyl ether fraction > Petroleum fraction (Saravanan et al., unpublished data). Therefore, the present study has been undertaken to evaluate the anti-atherogenic activity of ethanolic fractions of *T. arjuna* bark powder in high fat diet (HFD) fed rabbits.

## 2. Methods

### 2.1. Plant Materials

*T. arjuna* supercritical extract was procured from Elles Pvt. Ltd, Chennai, India, and authenticated by the Department of Botany at the Centre for Advanced Research, Indian System of Medicine (CARISM), SASTRA University, Thanjavur, India. A voucher specimen (No. 0066) has been deposited in the same department.

### 2.2. Fractionation of *T. arjuna*

Partial partition method was adapted to fractionate the *T. arjuna* bark powder. In total, 1500 g of supercritical bark powder was soaked in Petroleum ether for 7 days to defatting. On the 8th day, the petroleum ether soluble matters were decanted and concentrated under the reduced pressure. Subsequently, every 7th day consecutively, the fractionation was done similarly with different solvents from diethyl ether (TA-01), ethyl acetate (TA-02) and ethanol (TA-03) in the same extract powder as described by Row et al. [13]. All the fractions were dried under reduced pressure and the brown color precipitate was separated out. Fingerprinting of all fractions (TA-01, TA-02 and TA-03) was done by HPTLC-based method (data not shown). HPTLC fingerprinting of these fractions of TA were carried out using Linomet V spotter and scanned on TLC scanner-II (CAMAG with Cats 3.18 software) using silica gel 60 F_254_ TLC plates (Merck). The solvent system used was chloroform:methanol (9 : 1) and toluene : ethyl acetate:methanol : formic acid (5 : 4 : 1 : 0.5) and the plate was scanned at 366 nm. The peaks of three fractions observed were compared with each other. Further, the fractions were screened with GC-MS analysis and matched with Wiley & NIST system Library (Perkin Elmer : Clarus 500).

### 2.3. Phytochemical Screening

Phytochemical screening was performed on fractions TA-01, TA-02 and TA-03 to test the presence of phenolics compounds, tannins, glycosides, saponins, alkaloids and flavonoids in the bark of *T. arjuna* using appropriate tests [14].

### 2.4. Animals and Diet

All experiments were performed on female Swiss albino mice weighing 25–30 g and white New Zealand rabbits, both male and female, weighing 1.5–2 kg [15] obtained from Central Animal Facility (CAF), SASTRA University, Thanjavur. Animals were housed individually in polypropylene cages (mice) and stainless steel cages (rabbits) and kept in a room maintained at an average temperature of 22°C ± 3°C and humidity 55.6%, with 12 h darkness (lights on from 06:30 to 18:30 h) and fed with standard pellet diet (Tetragon Pvt. Ltd, Bangalore, India) before a week of start of experiment. The mice and the rabbits were allowed to have free access to food and tap water *ad libitum*. From then on (*t* = 0) until the end of the experiment, rabbits were fed a fat rich-chow (Table 1) obtained from Tetragon Pvt. Ltd, Bangalore, India. The animals were kept in cages with raised floors of wide wire mesh to prevent coprophagy. All experiments were performed in accordance with the protocol approved by the CAF and institutional ethical committee (No: 27/SASTRA/IAEC/RPP), SASTRA University, Thanjavur.

### 2.5. Acute Oral Toxicity Study

In an acute toxicity study, using the up- and down-procedure, all fractions in peanut oil (OECD 425; Accepted vehicle) were administered by oral gavage to female Swiss albino mice. The mice were *∼*6 weeks old and weighed from 25 to 30 g. The general procedure was as follows: one mouse was dosed at 175 mg kg^−1^ body weight (b.w.) and if no mortality or overt toxicity occurred within 48 h, another mouse was dosed at 550 mg kg^−1^ b.w. In the absence of toxicity, a third mouse was dosed at 2000 mg kg^−1^ b.w., and, if again no evidence of toxicity was observed, two additional mice were dosed at this level, that is, 2000 mg kg^−1^ b.w. [16]. In all cases, the dosing volume is fixed at 10 ml kg^−1^ b.w. The mice were housed individually in Polypropylene cages and were observed for clinical signs of toxicity at 0–0.5, 0.5–1, 1-2, 2–4 and 48 h post dosing (with special attention during the first 4 h). The body weights of each mice were recorded prior to test articles administration and at 7 and 14 days post dosing. Once daily, cage-side observations included changes in skin fur, eyes and mucous membrane (nasal) and also respiratory rate, circulatory (heart rate and blood pressure), autonomic (salivation, lacrimation, perspiration, piloerection, urinary incontinence and defecation) and central nervous system (ptosis, drowsiness, gait, tremors and convulsion) changes. The blood plasma was collected through retro orbital sinus (1 ml) for the hematological assessments. The mice were sacrificed by high dose of Ketamine HCl, intravenous (anesthetic dose: 22 mg kg^−1^ intramuscular) on 14 days post-dosing. The time of death, if any, was recorded. Necropsy included a gross examination of all major organs. The study was conducted in compliance with OECD Test Guideline 425 (Revised: 17 December 2001).

### 2.6. Selection of Dose

LD_50_ was done as per OECD guidelines for fixing the dose for biological evaluation. The LD_50_ of ethanolic fraction of TA falls under class four values with no signs of acute toxicity at 2000 mg kg^−1^ in mice. For biological evaluation in rabbits, the mice LD_50_ dose was converted into rabbit dose based on the body surface area and metabolic rate constant (FDA Guidelines, 2002). Hence, it was found that 926.66 mg kg^−1^ and from which the biological evaluation was carried out at approximate doses of 100 and 200 mg kg^−1^ in rabbits.

### 2.7. Experimental Design

Preceding the study, all rabbits consumed the same diet for one week. Twenty New Zealand rabbits were randomly divided into five groups each containing four animals of both sexes (1:1) and fed one of the following for 72 days: normal diet (shown above, *n = *4); HFD (*n = *4); HFD with standard drug Atorvastatin (1 mg kg^−1^, *n* = 4); HFD with EtOH fraction dose I (100 mg kg^−1^, *n = *4); HC with EtOH fraction dose II (200 mg kg^−1^, *n = *4). The rabbit were weighed every week. At the end of experiment, all rabbits were deprived of food overnight and sacrificed under lethal dose of sodium pentobarbital 100 mg kg^−1^ b.w. Confirmed death by the absence of a heart-beat, using thoracic auscultation. Blood was collected, and whole blood and serum were prepared for laboratory analysis. The major organs and aorta of each rabbit were harvested, washed with ice-cold isotonic saline and weighed. The serum samples were stored in refrigerator and aorta samples were stored in deep freezer until analyzed.

### 2.8. Serum Lipid Profile

Blood samples were taken from the rabbits on baseline, 14th day, 36th day and 72nd day and the serum was used to determine levels of total cholesterol (TC), triglyceride (TG), low density lipoprotein cholesterol (LDL-C), very low density lipoprotein cholesterol (VLDL-C) and high density lipoprotein cholesterol (HDL-C). The concentration of the above-said parameters is measured using biochemical test kits with the semi auto analyzer by colorimetric method.

### 2.9. Atherogenic Index

The atherogenic index serum (AIS) which is the measure of the extent of atherosclerotic lesions based on serum lipids is determined in all five groups. The atherogenic index is calculated using the formula AIS = TC/HDL [17].

### 2.10. Processing of Aorta Fragment

After the rabbits were sacrificed, tissues between their origin and bifurcation into the iliac arteries were taken gently, and washed with ice cold sterile physiologic saline to remove debris and blood residues. The proximal ascending aortic arch was dissected from each rabbit and divided into three 3-mm cross sections. These cross sections of aorta were cut open longitudinally and the adventitial coat was removed and the tunia media and tunia intima were stored in deep freezer (−80°C) for biochemical analyses [18].

### 2.11. Assessment of Atherosclerotic Plaques

Staining of a paraffin section with Haematoxylin (Hx) and Eosin (E) was done according to the Carleton method. The aorta was removed from the formal saline and a block of hard paraffin with the tissue in its center was prepared for sectioning. The paraffin (wax around the organ) was dissolved by putting the slide in xylol solution for 3 min, and then the xylol was replaced by alcohol by putting the slide in absolute alcohol. The slide was hydrated by putting it in descending grades of alcohol (in 100% alcohol, then in 90%, then in 70% alcohol and finally distilled water) for 3 min in each step. The section was stained in Hx for 7 min. This basic stain stains the nuclei and the basophilic structures of the cytoplasm with a blue color. The slide was put in tap water for 5 min to blue the section since the nuclei appears darker after tap water. The section was stained in E for 1 min. Eosin stains the acidophilic structures of the cytoplasm with red color. The slide was then washed in distilled water for 3 min. Dehydration of the slide was done by putting it in ascending grades of alcohol (for 1 min in 70% alcohol, then, for 3 min in 90% and for another 3 min in 100% alcohol). The slide was put in xylol in order to clear it from alcohol and to allow it to be miscible with Canada balsam. A drop of Canada balsam is put on a clean cover and the slide was removed from xylol with its face downwards and is put quickly on the cover. The stained section is thus ready for microscopical examination.

### 2.12. Statistical Analysis

All data are expressed as mean ± SEM and the statistical significance was performed by repeated measures ANOVA with Turkey's multiple comparison *post hoc* test. A *P*-value < .05 was considered significant.

## 3. Results

### 3.1. Fractionation and Phytochemical Investigation

**Figure 1:**
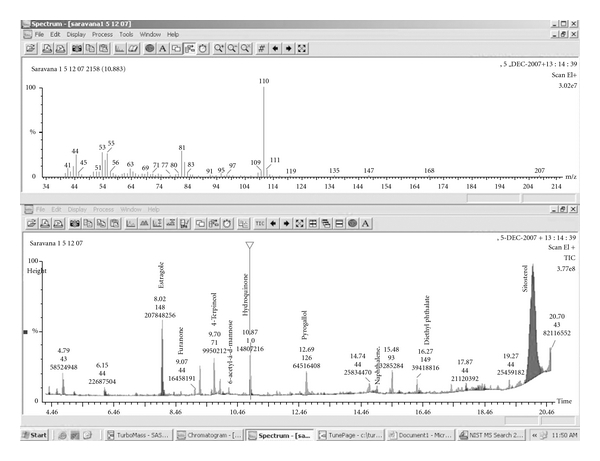
GC-MS analysis showing the presence of plant sterols.

The yields (Y) of the fractions TA-01, TA-02 and TA-03 were found to be 0.42, 0.978 and 24.16%, respectively. The yield of the ethanol fraction was much higher (i.e., 24.16%) than the other fractions. Phytochemical analysis revealed that TA fractions (TA-01, TA-02 and TA-03) contain phenolics, tannins, triterpenoid, saponins, anthraquinone glycosides, alkaloids and flavonoids. The HPTLC fingerprint combined with phytochemical analysis strongly suggests that the peaks observed correspond to phenolic compounds, tannins, glycosides, saponins, alkaloids and flavonoids [13, 19–21]. The GC-MS studies concluded that the presence of plant sterols is high in ethanolic fraction (Figure 1).

### 3.2. Acute Toxicity Studies

The acute oral toxicological study has not shown any deviation from the normal behavior of the Swiss albino mice during the entire study period. So, there were no acute toxicological changes for the ethanolic fraction of *T. arjuna* up to 2000 mg kg^−1^ b.w. of the animals. The biological evaluation was carried out at doses of 100 and 200 mg kg^−1^ b.w. in rabbits.

### 3.3. Lipid Profile

Total cholesterol concentrations of HFD group (baseline 53.42 ± 3.66 mg dl^−1^) were increased to 143.36 ± 8.01, 552.55 ± 42.00 and 868.43 ± 47.98 mg dl^−1^ after 14, 36 and 72 days, respectively. At the same time, Atorvastatin points out significantly lowered levels of TC to 49.91, 51.16 and 55.70% of those of the HFD (*P* < .05). Similarly, a significant reduction in the total cholesterol levels was also evident in HFD with EtOH fraction of *T. arjuna* bark. When treated with EtOH in 100 mg kg^−1^ of dose, the total cholesterol was reduced to the levels of 14.27, 27.61 and 30.36% of those of the HFD group (*P* < .05). Treated group of EtOH fraction in 200 mg kg^−1^, the TC level was found to have of reduction to 24.16, 37.91 and 44.41% of those of the HFD group (*P* < .05). Triglyceride concentrations of HFD group (70.62 ± 4.11) were increased to 259.76 ± 28.49, 565.50 ± 33.55 and 784.36 ± 47.92 after 14, 36 and 72 days, respectively. Atorvastatin significantly lowered the levels of TG to 57.1, 67.29 and 70.35% of those of the HFD (*P* < .05). When treated with EtOH fraction in 100 mg kg^−1^ of dose, the TG reduced levels to 19.43, 22.29 and 29.62% of those of the HFD group (*P* < .05). Treated group of EtOH fraction in 200 mg kg^−1^, the TG level was reduced to 35.36, 37.31 and 46.96% of those of the HFD group (*P* < .05). Changes in the LDL-C and VLDL-C levels were paralleled to serum cholesterol and triglycerides in all the treated groups and significant reduction in LDL-C and VLDL-C were found in Atorvastatin and EtOH fraction 100 and 200 mg kg^−1^ treated groups (*P* < .05) (Table 2). The HDL increased significantly in the treated group when compared with HFD group (*P* < .05) at 36 and 72 day of treatment. At 14th day treatment groups had shown no significant change in HDL level (*P* > .05).

### 3.4. Serum Atherogenic Index

**Figure 2:**
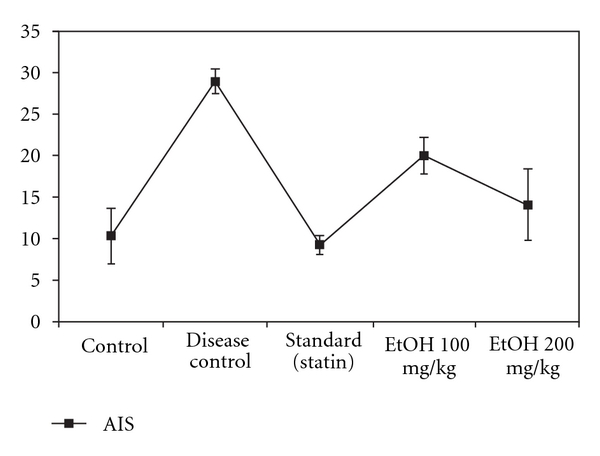
Atherosclerotic index.

Serum atherogenic index (28.94 ± 41.55) was high in HFD group after the 72 days of analysis and the significant reduction of atherogenic index was present in treated groups when compared with the HFD group (*P* < .05) (Figure 2).

### 3.5. Histopathological Findings

**Figure 3:**
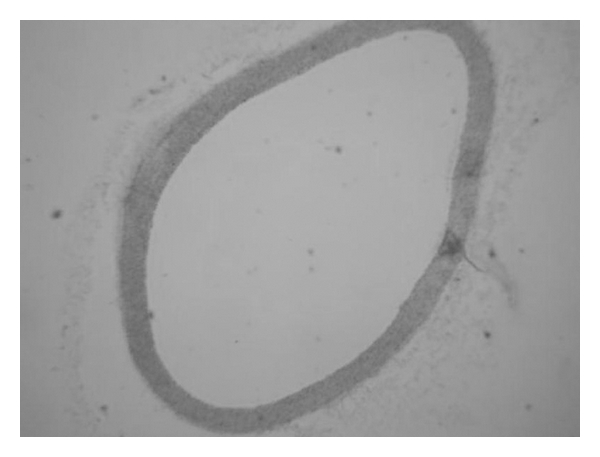
Aortic section of the control group rabbit revealing no pathological changes. HE × 5.

**Figure 4:**
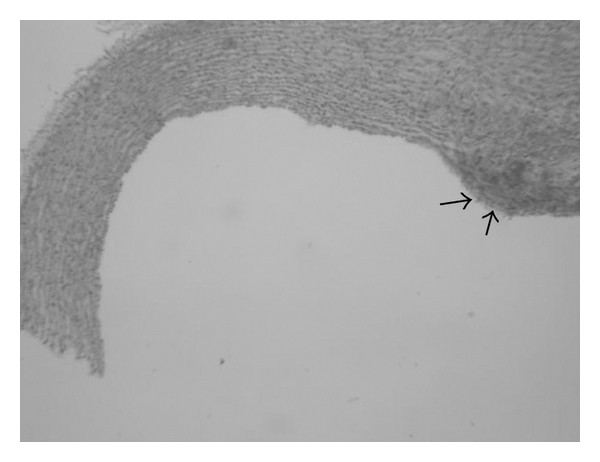
Thickening of the arterial wall in aortic section of the diseased control group. HE × 10.

**Figure 5:**
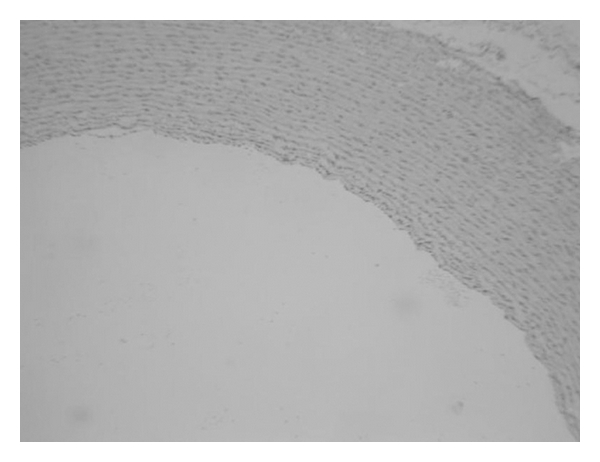
Presence of normal architecture of the aorta in the standard drug treated group. HE × 10.

**Figure 6:**
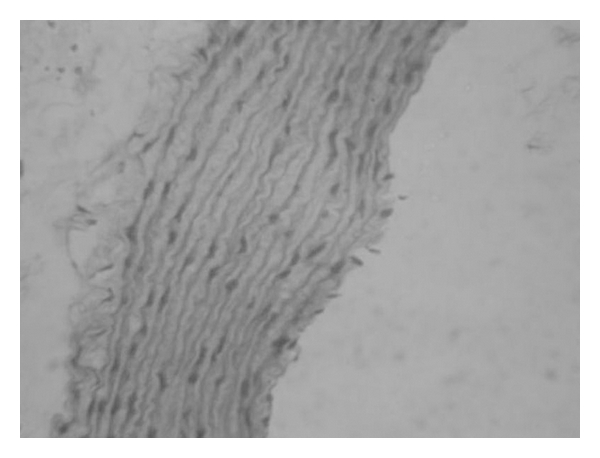
Low dose treated rabbit aortic section showing moderate thickening of the arterial wall. HE × 10.

**Figure 7:**
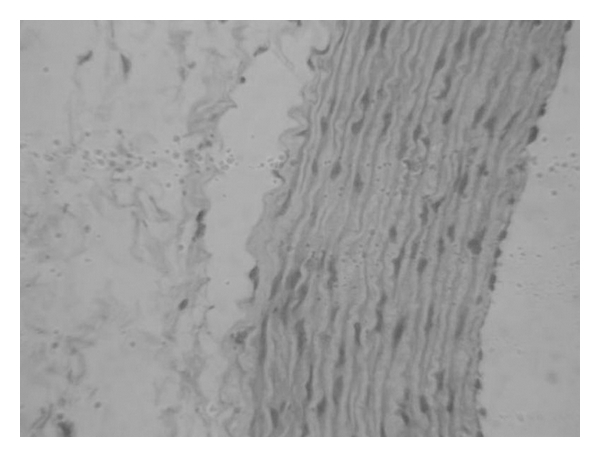
Aortic section of the high-dose group rabbit showing relatively normal arterial wall. HE × 40.

Histopathological examination of aorta obtained from the normal control group is shown in Figure 3. The aorta wall shows a uniform thickness with no bulging in the lumen and the endothelial lining is intact without any interruption. Also, elastic lamina and muscle fibers appear normal. On the other hand, all rabbits that received the 24% high fat-enriched diet for 72 days developed atherosclerotic lesions that appeared as marked alterations in the aortic wall, represented by the intimal plaques indicated by arrows in Figure 4. Administration of Atorvastatin in addition to a hypercholesterolaemic diet for 72 days completely improved the aortic architecture which is severely disturbed by hypercholesterolaemia. The aorta wall appears normal in Figure 5, with no foam cells, no disturbance in the endothelial lining and no change in the muscle fibers. In animals fed on high fat diet supplemented with EtOH fractions and high fat diet the tunica intima of the aorta is thickened but the atheromatous plaques are never as high and wide as plaques seen in the aorta of rabbits administered solely high fat diet. They are included with lipid droplets, foam cells and elastic fibers. Low dose treated rabbit aortic sections have indicated a moderate thickening of the arterial wall in all the rabbits (Figure 6). High dose treated rabbit aortic sections have revealed the presence of relatively normal aortic wall (Figure 7).

## 4. Discussion

**Figure 8:**
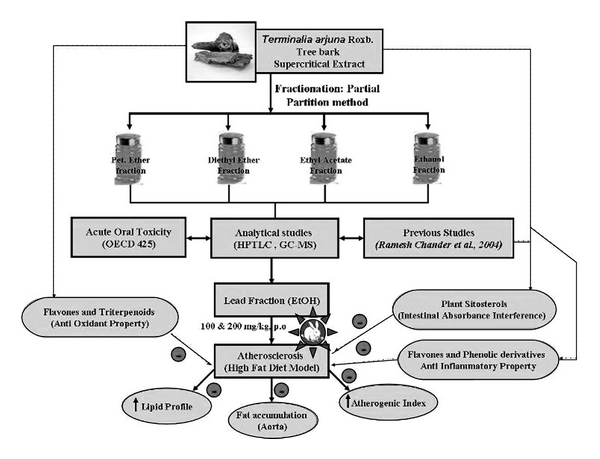
Schematic diagram.

Natural products extracts of therapeutic relevance are of paramount importance as reservoirs of structural and chemical diversity [22]. Epidemiological studies have reported a reduced risk of coronary heart disease in subjects with high flavonoid intake [23, 24]. The protective effect of flavonoids has been attributed to many mechanisms, that is, antioxidant properties, anti-inflammatory activity [25], antiproliferative as well as anti-platelet effects [26, 27]. The *T. arjuna* ethanolic fraction contains flavones and tannins [13, 28]. The GC-MS studies found that the presence of plant sterols is high in ethanolic fraction. The tannins and flavones may act as in free radical scavenging mechanism and may prevent atherogensis in rabbit aorta. While the plant sterols may be interacted with the intestinal absorption of fats and cholesterols, it will promote the fecal elimination of the fats and cholesterols [29]. The therapeutic activities of *T. arjuna* with the present study has been represented in a schematic diagram (Figure 8).

One of the key steps in the development of atherosclerosis is oxidative modification of LDL, which are scavenged by macrophages leading to formation of foam cells in the vessel wall, belonging to the early changes initiating the formation of atherosclerotic plaque. Atherosclerotic index indicates the deposition of foam cells or plaque or fatty infiltration or lipids in heart, coronaries, aorta, liver and kidney. The higher the atherosclerotic index the bigger the risk of these organs for oxidative damage. The Atherosclerotic index was significantly reduced in ethanolic fraction of *T. arjuna* treated hypercholesterolemic rabbits. BHUx-mediated reduction in the calcium content in the atherosclerotic plaque could also be attributed to its antioxidant property or to the calcium channel blocking property of *T. arjuna* [9].

A case-controlled study in rabbits fed on high cholesterol diet and administered *T. arjuna* bark powder 250 mg kg^−1^ twice daily was carried out recently to determine its hypolipidaemic effect. It was found that the rabbits receiving *T. arjuna* had a marked reduction in total cholesterol (*P* < .02) than control [27]. These findings are further confirmed in a later work [30–32].

In another experimental study this time using its ethanolic extract in doses of 100 and 500 mg kg^−1^, a significant reduction in total and LDL cholesterol are noted in hypercholesterolaemic rabbits. At 500 mg kg^−1^ doses also, a reduction is noted down in total: HDL and LDL:HDL ratio. It is also found that fat deposition in heart, liver and kidney is significantly low in those who received the drug. This has been related to our findings that the fat is deposited in the liver and heart. The absolute liver weight is increased and HFD groups expressed as increased weight and fatty liver. Significantly, the extract has not adversely affected the biochemical tests of liver and renal functions, and haematological parameters [33]. It relates our present report, that there was no significant change in hematological parameters (*P* > .05) except WBC. In HFD group, WBC count is increased significantly (*P* < .05) and the treated group shows the improvement in the WBC change (*P* < .05). In the present study, HDL shows enhancement on prolonged treatment of the EtOH fraction at high dose level (200 mg kg^−1^). Atorvastatin shows that significant (*P* < .05) enhancement in HDL at 14, 36 and 72 days of the study period. Observation that HDL cholesterol has not changed significantly are at variance to that of earlier works [30–32]. This result has been correlated to our study findings. Fluctuation in the HDL levels needs to be sorted out. Among three *Terminalias*, *T. arjuna* is observed to be the most potent hypolipidaemic agent. Interestingly, it has also raised high-density lipocholesterol. Besides hypolipidaemia, it has also induced partial inhibition of aortic atherosclerosis, thus showing anti-atherogenic properties [19]. *T. arjuna* is also highly effective in inhibiting cell proliferation and possesses anti-tumor activity through the interaction between transcription factors and target DNA sequences [34].

## 5. Conclusion

The present study suggests that the ethanolic fraction from *T. arjuna* bark powder has significant anti-atherogenic activity when administered to normal or hypercholesterolemic rabbits. The cardioprotective and anti-atherogenic effects of ethanolic fraction of *T. arjuna* may be due to presence of flavonoids, tannins and plant sterols. To elucidate the precise mechanism of action of specific biological moiety, further processing of ethanolic fraction of *T. arjuna* is required.

## Figures and Tables

**Table 1:** Composition of rabbit diet.

Compositions	Normal diet (%)	HFD (%)
Moisture	7.36	3.24
Crude protein	16.45	22.15
Crude fat	2.89	24.72
Crude fiber	12.24	3.6
Calcium	1.26	1.16
Phosphorous	0.85	0.86
Total ash	6.79	6.83

**Table 2:** Serum lipid profile and atherogenic index.

No. of days	Parameters	Control	D. Control	Std	EtOH 100 (mg kg^−1^)	EtOH 200 (mg kg^−1^)
Baseline						
	TC (mg dl^−1^)	54.02 ± 1.99	53.42 ± 3.66	55.84 ± 3.44	54.91 ± 4.50	54.38 ± 3.04
	HDL (mg dl^−1^)	31.69 ± 1.95	30.03 ± 1.15	29.47 ± 1.50	26.62 ± 1.88	30.62 ± 2.16
	LDL (mg dl^−1^)	7.50 ± 3.41	5.86 ± 3.89	10.93 ± 3.62	14.13 ± 4.54	9.64 ± 2.06
	VLDL (mg dl^−1^)	14.83 ± 0.73	17.53 ± 0.48	15.44 ± 0.77	14.16 ± 0.66	14.12 ± 0.82
	TG (mg dl^−1^)	74.15 ± 3.65	87.63 ± 2.42	77.18 ± 3.85	70.80 ± 3.31	70.62 ± 4.11
14 days						
	TC (mg dl^−1^)	63.63 ± 4.95	143.36 ± 8.01^(a)^	71.82 ± 5.30^(b)^	122.90 ± 8.18^(a),(b),(c)^	108.76 ± 6.67^(b),(c),(d)^
	HDL (mg dl^−1^)	35.13 ± 1.98	34.75 ± 4.27	38.25 ± 4.35	38.44 ± 1.62	40.50 ± 2.65
	LDL (mg dl^−1^)	7.09 ± 7.09	15.86 ± 12.92	6.14 ± 5.68	15.73 ± 4.36	13.69 ± 9.78
	VLDL (mg dl^−1^)	35.59 ± 1.66	92.75 ± 12.67^(a)^	39.70 ± 1.62^(b)^	68.73 ± 3.90^(a),(b),(c)^	51.95 ± 5.70^(a),(b),(d)^
	TG (mg dl^−1^)	177.93 ± 8.29	463.77 ± 63.37^(a)^	198.50 ± 8.10^(b)^	373.63 ± 19.48^(a),(b),(c)^	299.76 ± 28.49^(a),(b),(d)^
36 days						
	TC (mg dl^−1^)	254.86 ± 2.18	552.55 ± 42.00^(a)^	269.83 ± 11.45^(b)^	399.96 ± 41.61^(a),(b),(c)^	359.65 ± 41.08^(a),(b),(c)^
	HDL (mg dl^−1^)	33.94 ± 2.21	32.94 ± 3.56	38.00 ± 5.89	34.00 ± 4.40	37.91 ± 1.64
	LDL (mg dl^−1^)	170.49 ± 5.41	347.48 ± 32.80^(a)^	175.53 ± 9.05^(b)^	177.33 ± 39.96^(b)^	208.64 ± 42.99^(b)^
	VLDL (mg dl^−1^)	50.43 ± 6.57	172.13 ± 12.35^(a)^	56.29 ± 1.79^(b)^	138.63 ± 3.72^(b)^	113.10 ± 6.71^(a),(b),(c),(d)^
	TG (mg dl^−1^)	252.15 ± 32.84	860.65 ± 61.77^(a)^	281.46 ± 8.96^(b)^	663.16 ± 18.58^(a),(b),(c)^	539.50 ± 33.55^(a),(b),(c),(d)^
72 days						
	TC (mg dl^−1^)	353.78 ± 5.78	868.43 ± 47.98^(a)^	384.69 ± 4.94^(b)^	604.58 ± 27.42^(a),(b),(c)^	543.47 ± 53.91^(a),(b),(c),(d)^
	HDL (mg dl^−1^)	34.25 ± 1.71	30.00 ± 1.15	41.75 ± 4.92^(b)^	35.25 ± 2.99	38.58 ± 4.38^(b)^
	LDL (mg dl^−1^)	252.41 ± 7.47	582.44 ± 52.37^(a)^	267.06 ± 2.23^(b)^	489.17 ± 33.85^(a),(b),(c)^	348.02 ± 63.85^(a),(b),(d)^
	VLDL (mg dl^−1^)	67.11 ± 3.45	255.98 ± 7.82^(a)^	75.88 ± 2.25^(b)^	180.16 ± 13.48^(a),(b),(c)^	156.87 ± 9.58^(a),(b),(c),(d)^
	TG (mg dl^−1^)	335.57 ± 17.27	1279.92 ± 39.09^(a)^	379.38 ± 11.23^(b)^	900.79 ± 67.40^(a),(b),(c)^	704.36 ± 47.92^(a),(b),(c),(d)^
	Atherogenic Index	10.32 ± 2.38	28.94 ± 2.55	9.21 ± 1.02	19.98 ± 4.18	14.08 ± 3.31

Values expressed are mean ± SD of four animals per group.

^(a),(b),(c),(d)^statistical significance at *P* < .05 when compared with control, disease control, standard and treatment, respectively, on performing repeated measures ANOVA with Tukey's multiple comparison *post hoc* test.
